# Titanate-Coupled Aluminum as an Interfacial Modifier for Enhanced Thermal and Mechanical Performance in Hybrid Epoxy Composites

**DOI:** 10.3390/polym17141922

**Published:** 2025-07-11

**Authors:** Hai-Long Cheng, Seul-Yi Lee, Na Chu, Se-Yeol Lee, Fan-Long Jin, Soo-Jin Park

**Affiliations:** 1 Department of Polymer Materials, Jilin Institute of Chemical Technology, Jilin 132022, China; chl_111@126.com; 2 Department of Mechanical Engineering, College of Engineering, Kyung Hee University, Yongin 17104, Republic of Korea; leesy1019@inha.ac.kr (S.-Y.L.); ll1213856789@gmail.com (N.C.); 3 Department of Chemistry, Inha University, Incheon 22212, Republic of Korea; lsy2310@inha.edu

**Keywords:** DGEBA, expanded graphite, aluminum nanoparticles, thermal conductivity, impact strength

## Abstract

Thermally conductive polymer composites are essential for effective heat dissipation in electronic packaging, where both thermal management and mechanical reliability are critical. Although diglycidyl ether of bisphenol-A (DGEBA)-based epoxies exhibit favorable properties, their intrinsically low thermal conductivity limits broader applications. Incorporating conductive fillers, such as expanded graphite (EG) and metal powders, enhances heat transport but often compromises mechanical strength due to poor filler–matrix compatibility. In this study, we address this trade-off by employing a titanate coupling agent to surface-modify aluminum (Al) fillers, thereby improving interfacial adhesion and dispersion within the DGEBA matrix. Our results show that incorporating 10 wt% untreated Al increases thermal conductivity from 7.35 to 9.60 W/m·K; however, this gain comes at the cost of flexural strength, which drops to 18.29 MPa. In contrast, titanate-modified Al (Ti@Al) not only preserves high thermal conductivity but also restores mechanical performance, achieving a flexural strength of 35.31 MPa (at 5 wt% Ti@Al) and increasing impact strength from 0.60 to 1.01 kJ/m^2^. These findings demonstrate that interfacial engineering via titanate coupling offers a compelling strategy to overcome the thermal–mechanical trade-off in hybrid composites, enabling the development of high-performance materials for advanced thermal interface and structural applications.

## 1. Introduction

The relentless miniaturization and functional integration of microelectronic devices have led to a substantial increase in heat generation per unit volume, posing significant challenges to maintaining device stability and long-term reliability. Efficient thermal management has thus become a critical concern, particularly in high-frequency and high-power electronic systems, where localized temperature rises can accelerate materials degradation and lead to the failure of internal circuits [1,2,3,4].

Thermal interface materials (TIMs) are pivotal in mitigating these thermal issues by facilitating effective heat transfer between heat sources and sinks. An ideal TIM should exhibit not only high thermal conductivity but also maintain mechanical integrity, electrical insulation, and environmental stability, particularly when integrated into compact device architectures [5,6,7,8].

Epoxy resins have been extensively utilized as matrix materials for TIMs due to their favorable mechanical strength, low density, chemical resistance, dimensional stability, and ease of processing [9,10,11,12]. Their compatibility with various substrates and ability to encapsulate or adhere to components make them attractive in electronics packaging applications. However, the inherently low thermal conductivity of epoxy resins, typically less than 0.2 W/m·K, renders them insufficient for modern heat-intensive systems [13,14,15,16,17,18].

To enhance the thermal conductivity of epoxy systems, the incorporation of thermally conductive fillers has been a focal point of research [19]. These fillers aim to establish conductive pathways within the polymer matrix through percolation or bridging mechanisms, effectively reducing thermal resistance. Various filler types, including carbon-based materials (e.g., graphene, carbon nanotubes, graphite) [20,21,22,23,24], metal particles (e.g., silver (Ag), copper (Cu), aluminum (Al)) [25,26,27], and ceramic phases (e.g., aluminum nitride (AIN), boron nitride (BN), aluminum oxide (Al_2_O_3_)) [28,29,30], have been investigated for this purpose.

Among these, expanded graphite (EG), obtained by thermal exfoliation of natural graphite flakes, stands out due to its high in-plane thermal conductivity, low density, and excellent processability [31]. EG’s porous and worm-like structure, enriched with functional groups at the edges, enhances interfacial interaction and dispersion within epoxy matrices, enabling the formation of effective thermal conduction networks even at moderate filler contents [32,33,34,35].

Metal fillers, such as Al, offer high intrinsic thermal conductivity (~237 W/m·K), cost-effectiveness, and abundant availability, making them attractive for scalable thermal composites [36,37]. However, their practical use is hindered by poor compatibility with organic matrices due to spontaneous surface oxidation. This resultant oxide layer increases interfacial thermal resistance and diminishes filler–matrix adhesion, adversely impacting mechanical and thermal performance. Additionally, Al powders, particularly in nano- or submicron form, tend to agglomerate, leading to heterogeneous filler distribution and void formation, which further compromise composite performance [38,39,40].

To address these challenges, surface functionalization of Al particles is essential to improve interfacial bonding, enhance dispersion, and promote network connectivity within the polymer [41]. Surface treatment of metallic fillers using coupling agents—such as organosilanes, titanates, and rare-earth oxides—has been reported to modify surface chemistry and introduce functional groups that react or physically adsorb onto the epoxy matrix [42,43,44,45]. These modifications improve filler–matrix compatibility, lower interfacial thermal resistance, and enhance filler dispersion, leading to improved thermal conductivity and mechanical reinforcement [46,47,48,49]. Notably, titanate coupling agents have shown potential in promoting organic–inorganic interfacial bonding by forming flexible, chemically anchored interphases that contribute to energy dissipation and load transfer under thermal and mechanical stress [50,51,52,53].

In this study, we report the design and fabrication of hybrid epoxy composites composed of diglycidyl ether of bisphenol-A (DGEBA) resin filled with expanded graphite and surface-modified aluminum powder. Aluminum particles were functionalized using isopropyl dioleic (dioctylphosphate) titanate to enhance dispersion and matrix compatibility. The composites were prepared via melt blending and compression molding to ensure uniform filler distribution and promote thermally conductive network formation. A comprehensive investigation was conducted to evaluate the effects of Al surface modification on the thermal conductivity, thermal stability, mechanical strength, and microstructural characteristics of the DGEBA/EG/Al composites. In the present work, DGEBA/EG/Al hybrid composites were prepared using high-aspect-ratio carbon fillers and chemically tailored metal particles, which solved the problem of low thermal conductivity of epoxy resin and provided a synergistic approach for lightweight and thermally conductive epoxy resin systems, suitable for next-generation electronic packaging and heat dissipation applications. The hybrid epoxy composites prepared in this study, such as TIMs, can be widely used in devices that require efficient heat dissipation or uniform temperature, such as smart phones, laptops, LED car lights, electric vehicle battery packs, laser equipment, and 3D printers.

## 2. Experimental

### 2.1. Materials

**Figure 1:**
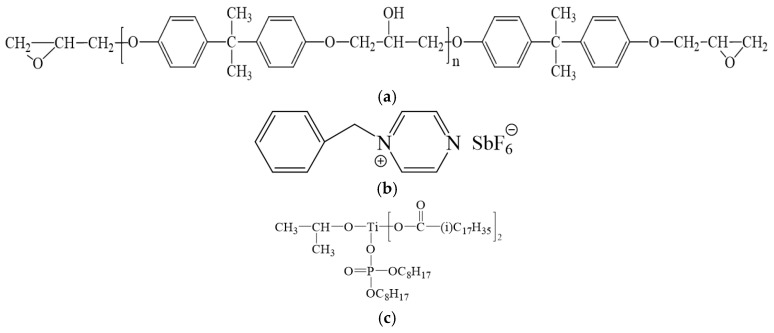
Chemical structures of (**a**) DGEBA, (**b**) BPH, and (**c**) the titanate coupling agent.

**Figure 2:**
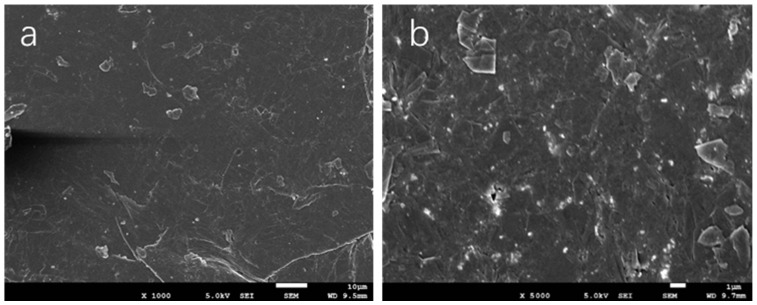
SEM images of EG: (**a**) magnification of 1000, scale bar of 10 μm, (**b**) magnification of 5000, scale bar of 1 μm.

The DGEBA was obtained from Nantong Xingchen Synthetic Material Co., Ltd. (Nantong, China), which has an epoxy equivalent weight of 184−195 g/mol. Thermally latent initiator of the DGEBA epoxy resin *N*-benzylpyrazinium hexafluoroantimonate (BPH) was synthesized according to a previous report [54]. EG was supplied by Jiangxi Shuobang New Material Technology Co., Ltd. (Fuzhou, China), Aluminum powder was obtained from Tianjin Zhongxin New Material Co., Ltd.(Tianjin, China), which has a particle size of 50 nm. Isopropyl dioleic(dioctylphosphate) the titanate coupling agent was obtained from Guangzhou City Building Double Chemical Technology Co., Ltd. (Guangzhou, China). Anhydrous ethanol was purchased from Sinopharm Chemical Reagent Co., Ltd. (Shanghai, China). All the chemicals were of analytical grade and were used without further purification. The chemical structures of DGEBA, BPH, and the titanate coupling agent are shown in Figure 1. Figure 2 SEM images of EG at magnifications of 1000 and 5000.

### 2.2. Surface Modification of Al Powder

**Figure 3:**
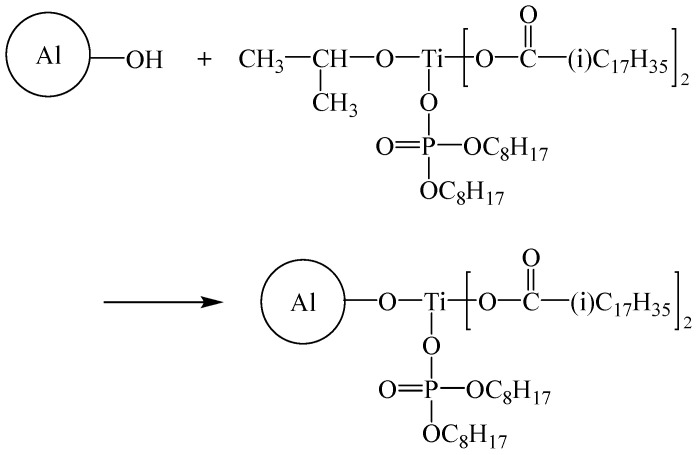
Scheme of surface modification of Al powder using the titanate coupling agent.

Titanate coupling agent (20 g) dispersed in anhydrous alcohol (100 mL) to obtain the titanate coupling agent-dispersed alcohol solution. Al powder (30 g) was added into the solution, and the mixture was mixed for 1 h using stirrer and ultrasonic treated for 1 h. The mixture was vacuum-filtered at 35 °C for 3 h, washed with deionized water several times, and dried at 60 °C for 3 h to produce the titanate coupling agent-modified Al nanoparticles. A schematic illustration of surface modification of Al powder using the titanate coupling agent is shown in Figure 3. After the surface modification, organic alkyl groups on the Al surface interact with the epoxy matrix, enhancing the compatibility of the filler with the matrix. For convenience, the titanate coupling agent-modified Al is labeled as Ti@Al in this paper.

### 2.3. Preparation of the Composite Samples

**Figure 4:**
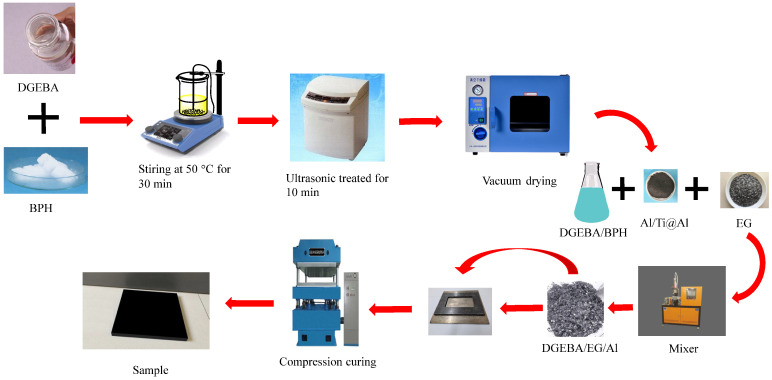
Schematic illustration of the preparation of DGEBA/EG/Al and DGEBA/EG/Ti@Al composites.

The preparation of the DGEBA/EG/Al and DGEBA/EG/Ti@Al composites is schematically shown in Figure 4. The EG content was 60 wt%, and the Al/Ti@Al content was varied from 0 to 10 wt%. The amount of BPH was 1 phr (parts per hundreds of epoxy resin). The DGEBA, EG, Al/Ti@Al, and BPH were added to a mixer and mixed at 80 °C for 30 min. Then, the mixture was added to a mold, which is preheated and sprayed with a mold release agent. The epoxy composites were polymerized at temperatures of 120, 160, and 190 °C and a pressure of 5 MPa for 1 h.

### 2.4. Characterization and Measurements

Fourier transform infrared (FTIR) spectra of pristine Al and Ti@Al were measured with a spectrometer (Tensor II, BRUKER Company, Ettlingen, Germany). The surface properties of Al and Ti@Al were investigated via X-ray photoelectron spectroscopy (XPS; Thermo ESCALAB 250, Waltham, MA, USA). The surface morphologies of Al and Ti@Al were obtained using scanning electron microscopy (SEM; S4800, Hitachi, Tokyo, Japan). Energy-dispersive X-ray spectroscopy (EDX) and SEM were performed to evaluate the content of the titanate coupling agent on the EG surface. The thermal conductivities of the DGBA/EG/Al and DGBA/EG/Ti@Al composites were investigated via a thermal conductivity tester (LFA467, Netzsch, Netzsch, Germany) according to the GB/T 10294-2008 standard [55]. Each sample size was 12.7 mm in diameter and 5 mm in thickness. Thermal conductivity values were calculated by averaging three experimental values. Thermal stabilities of the composites were investigated using thermogravimetric analysis (TGA; TA Instruments, Q50, New Castle, DE, USA) between 30 and 800 °C at 10 °C/min and N_2_ atmosphere. The flexural strength of the composites was evaluated using a mechanical testing apparatus (WDW 3010, Jinan, China) following the GB/T 9341-2008 standard [56]. The flexural strength (*σ_f_*) values were obtained by the following equation:

(1) $$\sigma_{f}=\frac{3PL}{2bd^{2}}$$

where P is the applied load, L is the span length, b is the width of the specimen, and d is the thickness of the specimen. The flexural strength values were calculated by averaging five experimental values.

The impact strengths of the composites were investigated via an Izod impact tester (TP04G-AS1, Dongguan, China) following the GB/T 1843-2008 standard [57]. The impact strength values were calculated by averaging five experimental values. The morphologies of the composites were examined via field-emission SEM (FE-SEM, JEOL, JSM-7610F Plus, Tokyo, Japan).

## 3. Results and Discussion

### 3.1. Characterization of Ti@Al

**Figure 5:**
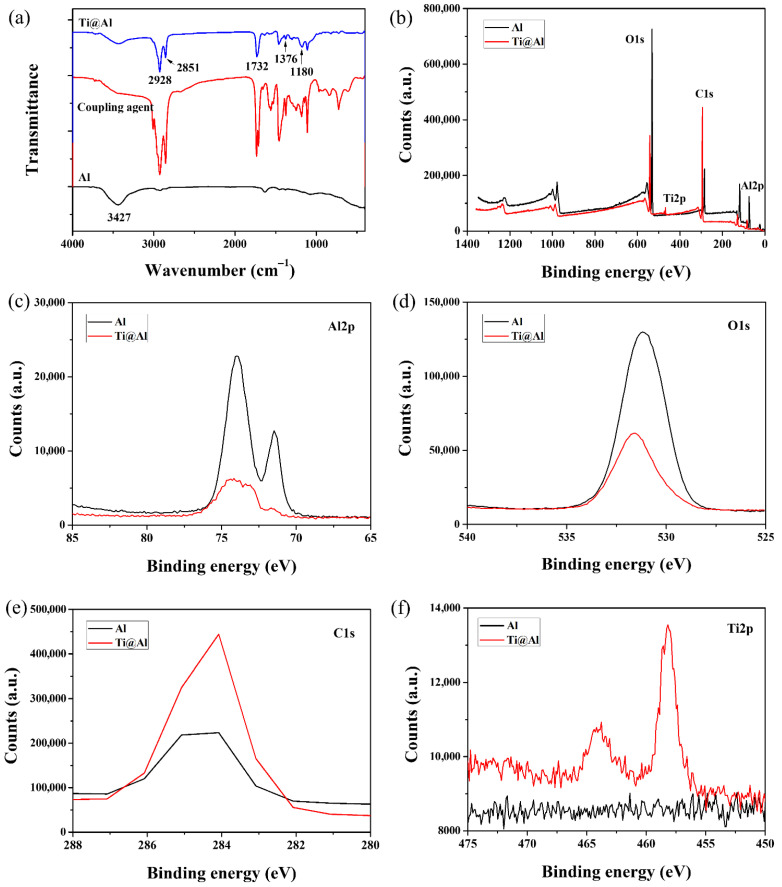
(**a**) FTIR spectra of Al and Ti@Al. (**b**–**f**) XPS spectra of Al and Ti@Al: (**b**) survey, (**c**) high-resolution Al2p, (**d**) high-resolution O1s, (**e**) high-resolution C1s, (**f**) high-resolution Ti2p.

The surface of Al powder was modified using isopropyl dioleyl (dioctyl phosphate) the titanate coupling agent, and the functional group variations in Al and Ti@Al were investigated using FTIR. Figure 5a shows the FTIR spectra of pristine Al, the titanate coupling agent, and Ti@Al. Pristine Al shows a characteristic absorption peak of the hydroxyl group at 3427 cm^−1^, and the intensity of the hydroxyl group decreased after the surface modification. After the surface treatment, two characteristic absorption peaks observed at 2928 and 2851 cm^−1^, which are attributed to the CH antisymmetric and symmetric stretching vibrations, respectively, and three characteristic absorption peaks observed at 1733, 1376, and 1180 cm^−1^, which are assigned to the C=O telescopic vibration, methyl symmetric bending vibration, and C−O stretching vibration, respectively [58]. These are because the titanate coupling agent reacts with hydroxyl group on the Al surface to introduce methylene, methyl, and C=O functional groups on the Al surface after the surface treatment [59,60].

The surface properties of pristine Al and Ti@Al were measured by XPS analysis, and XPS spectra are shown in Figure 5b–f. As shown in Figure 5c, the characteristic peaks of Al_2p_ appeared at 74 and 71.5 eV, and the intensity of Al_2p_ peaks decreased after the surface modification. The peak of O_1s_ appeared at 531.2 eV, and the O_1s_ peak intensity decreased after the surface modification, as shown in Figure 5d. In contrast, the peak of C_1s_ appeared at 284.1 eV, and the C_1s_ peak intensity increased after the surface modification, as shown in Figure 5e. Furthermore, two new peaks appeared at 464 and 458.2 eV, which are assigned to the titanium, as shown in Figure 5f [61,62]. In addition, the atomic ratio of C/O calculated from the XPS spectra increased from 0.24 for pristine Al to 1.45 for Ti@Al, which was due to the introduction of the long-chain alkyl group after the surface modification.

**Figure 6:**
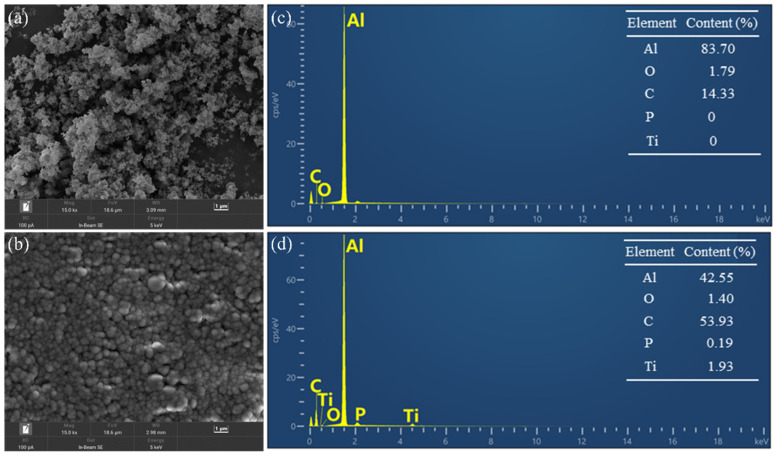
(**a**,**b**) SEM images of (**a**) Al and (**b**) Ti@Al (magnification of 150,000). (**c**,**d**) EDX maps of (**a**) Al and (**b**) Ti@Al.

To investigate the morphology of pristine Al and Ti@Al and to assess the content of the titanate coupling agent on the Al surface, SEM−EDX observations were carried out. Figure 6a,b show the morphologies of pristine Al and Ti@Al. Pristine Al exhibited an aggregated surface (Figure 6a). In contrast, Ti@Al showed a well dispersed surface coated with a thin layer, as shown in Figure 6b.

Figure 6c,d show the EDX maps of pristine Al and Ti@Al. As shown in Figure 6c, the peaks at 0.3, 0.5, and 1.5 keV can be assigned to carbon, oxygen, and aluminum, respectively. After the surface modification, two new peaks appeared at approximately 2.1 and 4.5 keV, which are assigned to the phosphorus and titanium, respectively (Figure 6d). Specially, after the surface treatment, aluminum and oxygen contents decreased from 83.7% and 1.97% to 42.55% and 1.4%, respectively; carbon, phosphorus, and titanium contents increased from 14.33%, 0%, and 0% to 53.93%, 0.19%, and 1.93%, respectively. The above results verify that the titanate coupling agent was successfully coated on the Al surface [63].

### 3.2. Thermal Conductivity

**Figure 7:**
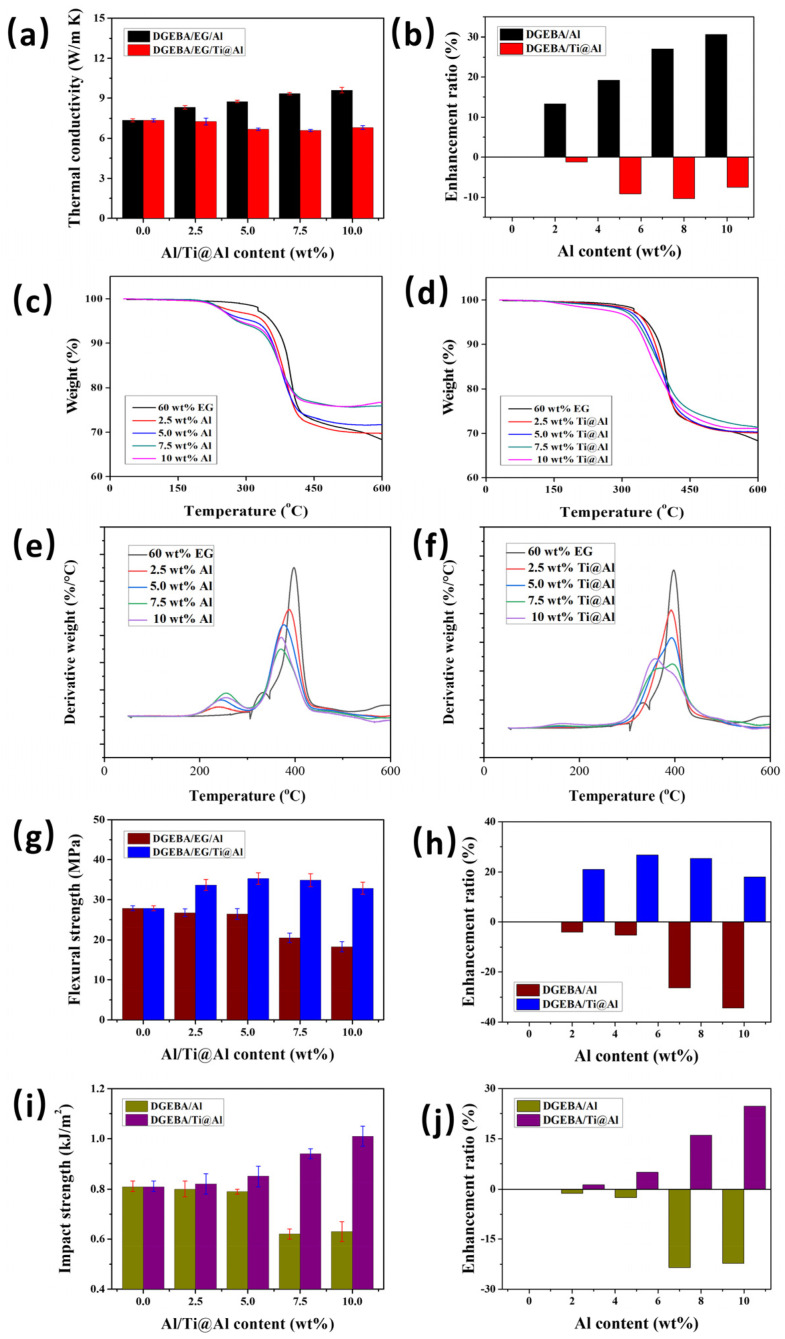
(**a**,**b**) (**a**) Thermal conductivity and (**b**) thermal conductivity enhancement ratio of DGEBA/EG/Al and DGEBA/EG/Ti@Al composites as a function of Al/Ti@Al content. (**c**,**d**) TGA thermograms of (**c**) DGEBA/EG/Al and (**d**) DGEBA/EG/Ti@Al composites as a function of Al/Ti@Al content. (**e**,**f**) DTA thermograms of (**e**) DGEBA/EG/Al and (**f**) DGEBA/EG/Ti@Al composites as a function of Al/Ti@Al content. (**g**,**h**) (**g**) Flexural strength and (**h**) flexural strength enhancement ratio of DGEBA/EG/Al and DGEBA/EG/Ti@Al composites as a function of Al/Ti@Al content. (**i**,**j**) (**i**) Impact strength and (**j**) impact strength enhancement ratio of DGEBA/EG/Al and DGEBA/EG/Ti@Al composites as a function of Al/Ti@Al content.

Table 1 and Figure 7a,b illustrate the thermal conductivity trends and enhancement ratios for DGEBA/EG/Al and DGEBA/EG/Ti@Al composites as a function of Al and surface-modified Ti@Al filler content. The thermal conductivity of the DGEBA/EG/Al composites shows a clear upward trend with increasing Al content. The reference DGEBA composite containing 60 wt% EG alone exhibited a thermal conductivity of 7.35 W/m·K. Upon incorporating 10 wt% of unmodified Al powder, the thermal conductivity increased markedly to 9.60 W/m·K, corresponding to an enhancement of approximately 31%.

This notable improvement is attributed to the role of Al fillers in bridging interfacial voids within the EG network. The porous, lamellar microstructure of EG inherently presents high interfacial thermal resistance due to trapped air and insufficient contact between adjacent flakes. The inclusion of Al nanoparticles effectively fills these voids and constructs a lamellar–spherical–lamellar hybrid network, facilitating phonon transport and the formation of continuous thermally conductive pathways throughout the epoxy matrix [61].

In contrast, the incorporation of Ti@Al led to a slight but consistent decline in thermal conductivity across all filler loadings. For instance, the thermal conductivity of the DGEBA/EG/Ti@Al composite with 7.5 wt% Ti@Al was 6.70 W/(m·K), which is approximately 9% lower than that of the DGEBA/EG composite containing only EG. This reduction is attributed to the formation of interfacial organic layers introduced by the titanate coupling agent during surface treatment. Specifically, the Al particle surfaces were coated with functional organic moieties such as methylene, methyl, and carbonyl (C=O) groups. These organic layers impede direct contact between Al particles and EG, thereby increasing the interfacial thermal resistance and disrupting electron and phonon transport across filler interfaces [64]. Consequently, the formation of a continuous, percolated thermal network is inhibited, resulting in a net reduction in thermal conductivity.

### 3.3. Thermal Stability

The influence of Al and surface-treated Ti@Al content on the thermal stability of DGEBA/EG-based composites was investigated using TGA and differential thermal analysis (DTA). The corresponding TGA and DTA thermograms are presented in Figure 7c–f, and key thermal stability parameters—namely the initial decomposition temperature (defined as the temperature at 5% weight loss, *T*_5%_), temperature at maximum weight loss rate (*T*_max_), and the char yield at 600 °C—are summarized in Table 2. These metrics were selected based on established criteria in thermal degradation analysis for polymer composites [65,66], and the thermal stability factors are listed in Table 2.

The *T*_5%_ value of the neat DGEBA/EG/Al composites (60 wt% EG) was measured at 355.7 °C. However, a progressive decrease in *T*_5%_ was observed with increasing unmodified Al content. At 10 wt% Al, the *T*_5%_ dropped markedly to 285.7 °C, indicating a significant 70 °C reduction relative to the control sample. This decline in thermal stability can be attributed to two primary factors: the inclusion of Al reduces the overall epoxy content in the composite, diminishing the number of reactive epoxide groups involved in the curing process and thereby lowering the crosslink density of the matrix. Second, as the Al content increases, particle agglomeration becomes more pronounced due to poor dispersion, which leads to increased matrix viscosity during processing, the formation of voids and large filler clusters, and insufficient interfacial bonding with the resin. These phenomena collectively compromise the thermal stability of the DGEBA/EG/Al composites [67].

Interestingly, the thermal degradation behavior of DGEBA/EG/Ti@Al composites exhibited a relatively enhanced thermal response compared to their unmodified counterparts under identical loading conditions. Although the *T*_5%_ values still decreased with the addition of Ti@Al, they remained 13–40 °C higher than those of the corresponding DGEBA/EG/Al composites. For instance, the *T*_5%_ at 7.5 wt% Ti@Al was recorded at 333.5 °C, compared to 279.9 °C for the equivalent Al-filled composite. This enhancement is primarily due to the improved dispersion and wettability of the Al particles after surface treatment with the titanate coupling agent. The introduction of organic functional groups (e.g., methylene, methyl, and carbonyl) onto the Al surface promotes better compatibility with the epoxy matrix, thereby reducing the occurrence of agglomeration and enhancing matrix-filler interactions during thermal decomposition. Similarly, although the *T*_max_ values decreased with the addition of Al/Ti@Al, the *T*_max_ values of DGEBA/EG/Ti@Al composites are higher than those of the DGEBA/EG/Al composites under the same conditions.

Additionally, the char yield at 600 °C increased with increasing filler content for both composite systems. This effect is mainly attributed to the high residual mass of Al, which does not undergo significant decomposition within the tested temperature range. The presence of such thermally stable fillers contributes positively to the flame resistance and structural integrity of the composite residue post-degradation.

### 3.4. Flexural Strength

The effects of Al and Ti@Al filler content on the flexural strength of DGEBA/EG-based composites were examined, with the results presented in Table 3 and Figure 7g,h. The neat DGEBA/EG composite exhibited a baseline flexural strength of 27.88 ± 0.61 MPa. However, the inclusion of untreated Al led to a consistent degradation in flexural performance. Specifically, at 5 wt% and 10 wt% Al content, the flexural strengths declined to 26.42 ± 1.36 MPa and 18.29 ± 1.27 MPa, respectively, corresponding to reductions of approximately 5% and 34% relative to the reference sample. This decline is primarily attributed to the poor dispersion and weak interfacial adhesion between the untreated Al particles and the epoxy matrix. As Al content increases, agglomeration becomes more pronounced, resulting in stress concentration zones and microstructural defects such as voids and interfacial delamination under bending loads [68]. These factors collectively compromise the stress transfer efficiency and structural integrity of the composite, especially at higher filler loadings.

In contrast, the incorporation of Ti@Al significantly improved the flexural properties of the composites. The DGEBA/EG/Ti@Al composite containing 5 wt% Ti@Al exhibited a flexural strength of 35.31 ± 1.46 MPa, representing a 27% improvement over the control sample without any Al additives. A further increase in strength was observed at 7.5 wt% Ti@Al (34.91 ± 1.61 MPa), with a slight decrease at 10 wt% Ti@Al (32.88 ± 1.51 MPa), although still significantly higher than the untreated counterpart.

The enhanced flexural performance of the Ti@Al-filled composites is attributed to the surface modification of Al particles using isopropyl dioleic (dioctyl phosphate) titanate, which grafts organic functional groups (e.g., methylene, methyl, C=O) onto the particle surface [69,70]. These groups improve chemical affinity between the filler and matrix, promoting stronger interfacial bonding and more homogeneous stress distribution under mechanical loading. Moreover, better dispersion of Ti@Al particles mitigates stress concentrations and reduces the formation of mechanical defects, thereby enhancing the composite’s load-bearing capacity.

### 3.5. Impact Strength

The impact strength behavior of DGEBA/EG/Al and DGEBA/EG/Ti@Al composites as a function of filler content is summarized in Table 4 and depicted in Figure 7i,j. These results reveal contrasting trends between the two composite systems depending on the surface treatment of the Al fillers. In the case of the DGEBA/EG/Al composites, the incorporation of Al powder consistently led to a reduction in impact resistance. The neat DGEBA/EG system exhibited an impact strength of 0.81 ± 0.02 kJ/m^2^. Upon addition of 5 wt% Al, this value declined slightly to 0.79 ± 0.01 kJ/m^2^, followed by a pronounced decrease to 0.63 ± 0.04 kJ/m^2^ at 10 wt% Al, marking an overall reduction of approximately 22%. This deterioration in impact performance is primarily attributed to poor filler dispersion and agglomeration of Al particles within the epoxy matrix at higher concentrations. Agglomerated metallic domains act as local stress concentrators, disrupting the continuity of the polymer matrix and facilitating crack initiation under mechanical loading [54,71]. Furthermore, the weak interfacial bonding between untreated Al particles and the epoxy resin hinders effective stress transfer, thereby amplifying brittleness and lowering energy absorption capacity during impact events.

Conversely, the DGEBA/EG/Ti@Al composites demonstrated a pronounced enhancement in impact resistance with increasing Ti@Al content. At 10 wt% Ti@Al, the impact strength reached 1.01 ± 0.04 kJ/m^2^, which represents a 25% increase compared to the reference composite. Unlike the flexural response, where improved interfacial stress transfer dominates, the improvement in impact performance is more strongly linked to energy dissipation via microstructural toughening mechanisms. Specifically, the surface modification of Al particles using titanate coupling agents improves their wettability and dispersion within the matrix, resulting in a more homogeneous microstructure. During impact, these well-dispersed Ti@Al fillers disrupt crack propagation paths and introduce localized heterogeneities that activate multiple deformation mechanisms, such as shear yielding and matrix tearing, rather than single-mode brittle fracture [72,73]. Furthermore, the interfacial zones formed by the organic functionalities act as semi-flexible buffer regions that absorb kinetic energy through interfacial sliding and matrix shear banding. This synergistic combination of particle dispersion and dissipative microstructural rearrangement substantially enhances the composite’s resistance to sudden, high-energy loading [74].

### 3.6. Morphology

**Figure 8:**
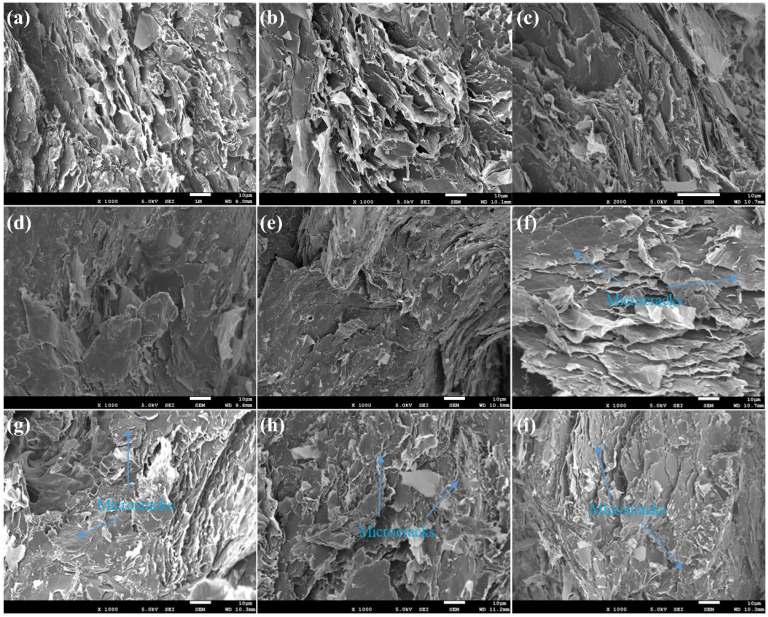
SEM micrographs of DGEBA/EG/Al and DGEBA/EG/Ti@Al composites: (**a**) 60 wt% EG, (**b**) 60 wt% EG + 2.5 wt% Al, (**c**) 60 wt% EG + 5 wt% Al, (**d**) 60 wt% EG + 7.5 wt% Al, (**e**) 60 wt% EG + 10 wt% Al, (**f**) 60 wt% EG + 2.5 wt% Ti@Al, (**g**) 60 wt% EG + 5 wt% Ti@Al, (**h**) 60 wt% EG + 7.5 wt% Ti@Al, (**i**) 60 wt% EG + 10 wt% Ti@Al (magnification of 1000).

**Figure 9:**
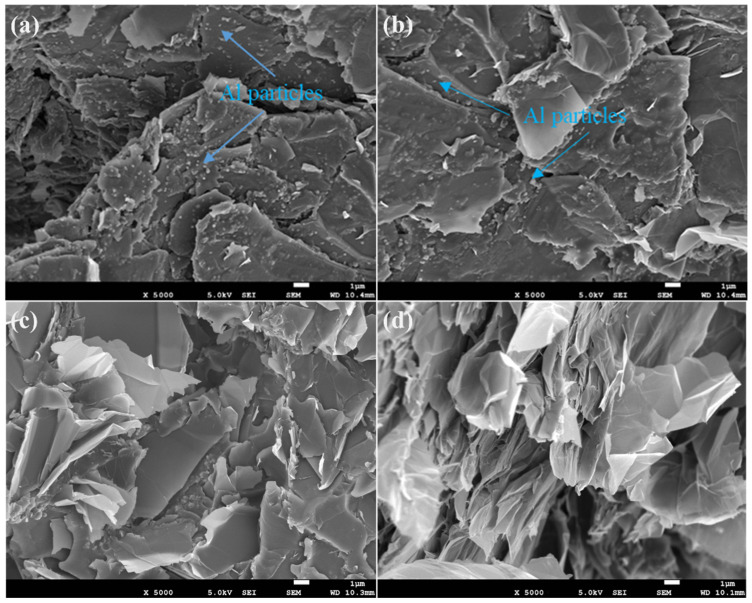
SEM micrographs of DGEBA/EG/Al and DGEBA/EG/Ti@Al composites at high magnification: (**a**,**b**) 60 wt% EG + 10 wt% Al, (**c**,**d**) 60 wt% EG + 10 wt% Ti@Al (magnification of 5000).

To gain insight into the fracture behavior and interfacial interactions of the composites, SEM measurement was carried out to observe the fractured surfaces of DGEBA/EG, DGEBA/EG/Al, and DGEBA/EG/Ti@Al composites following impact strength testing. Representative SEM images are presented in Figure 8, while high-magnification views of selected samples containing 10 wt% Al or Ti@Al are shown in Figure 9.

Figure 8a reveals that the DGEBA/EG composite exhibits a fracture surface characterized by the detachment of sheet-like graphite blocks, indicating delamination and localized brittle failure along EG-rich domains. This morphology suggests insufficient interfacial bonding between the graphite flakes and the epoxy matrix, consistent with moderate impact strength but enhanced in-plane thermal conductivity due to the formation of extended graphite domains [75]. In the DGEBA/EG/Al composites (Figure 8b–e), the overall morphology remains similar to that of the EG-only system, with graphite sheets and Al particles forming interpenetrating, yet discontinuous, thermally conductive pathways. The presence of Al particles between graphite layers contributes to improved thermal conductivity via lamellar–spherical–lamellar network formation. However, the fractured surfaces display increased delamination and larger sheet-like fragments, indicative of weak adhesion between Al, EG, and the DGEBA matrix.

In contrast, the fractured surfaces of DGEBA/EG/Ti@Al composites (Figure 8f–i) reveal a distinct morphological evolution. The delaminated graphite domains are markedly reduced, and the surface is populated with numerous fine microcracks distributed throughout the matrix. These microcracks are indicative of crack–bridging and crack–deflection mechanisms, which are known to enhance impact resistance by dissipating energy through controlled plastic deformation. This morphological signature aligns with the improved impact strength observed in Ti@Al-filled composites and confirms the role of surface modification in enabling better stress redistribution under dynamic loading [46].

High-resolution SEM images of composites with 10 wt% filler content (Figure 9) further elucidate the differences between untreated and surface-modified systems. In the DGEBA/EG/Al composite (Figure 9a,b), numerous unmodified Al particles are embedded within the matrix but are partially de-bonded or exposed, suggesting poor interfacial adhesion. These loosely bonded particles act as stress concentrators, facilitating interfacial crack initiation and propagation, which ultimately compromises the impact strength. On the other hand, the DGEBA/EG/Ti@Al composite (Figure 9c,d) exhibits a relatively smooth and cohesive fracture surface, with minimal evidence of filler pull-out. The absence of observable Ti@Al particles on the surface suggests strong interfacial adhesion and effective load transfer between the matrix and the surface-functionalized fillers. These findings confirm that the titanate surface modification significantly enhances the interfacial compatibility between Al particles and the epoxy matrix, leading to a more uniform filler dispersion and a reduction in structural defects such as agglomerates or voids. While this improved adhesion contributes to superior mechanical integrity and impact toughness, it simultaneously disrupts the formation of interconnected thermal pathways, thereby explaining the moderate reduction in thermal conductivity in Ti@Al-based composites. The contrasting microstructural features thus provide direct evidence for the trade-off between thermal and mechanical properties in hybrid epoxy composites and underscore the importance of interfacial engineering in tailoring performance based on application-specific requirements [76].

## 4. Conclusions

In this study, thermally conductive epoxy composites based on diglycidyl ether of bisphenol-A (DGEBA) were successfully fabricated using expanded graphite (EG) and either untreated or titanate-modified aluminum (Al and Ti@Al) fillers through hot blending and compression-curing processes. The effects of Al surface modification on the thermal, mechanical, and morphological properties of the resulting composites were systematically investigated. Surface functionalization of Al particles was verified via FTIR, XPS, SEM, and EDX analyses, confirming the successful grafting of organic groups that improve interfacial compatibility with the epoxy matrix. The incorporation of 10 wt% unmodified Al into DGEBA/EG composites enhanced thermal conductivity to 9.60 W/m·K, representing a 31% increase compared to the EG-only system, through the formation of lamellar–spherical–lamellar thermal conduction networks. In contrast, the DGEBA/EG/Ti@Al composites showed a 9% decrease in thermal conductivity due to the insulating nature of the surface modifier, which introduced additional interfacial thermal resistance. Thermal stability was negatively affected by the inclusion of Al, with the initial decomposition temperature (*T*_5%_) decreasing due to reduced crosslink density and filler agglomeration. However, Ti@Al-modified composites exhibited improved thermal degradation resistance relative to their unmodified counterparts, owing to enhanced filler dispersion and matrix–filler interactions. Mechanical testing revealed contrasting trends in flexural and impact performance. The flexural strength of DGEBA/EG/Al composites decreased from 27.8 to 19.0 MPa (a 31.7% reduction) as Al content increased to 10 wt%, due to poor filler dispersion and interfacial debonding. However, the addition of 5 wt% Ti@Al increased flexural strength to 35.3 MPa, a 27% improvement over the baseline, attributable to improved stress transfer from enhanced interfacial bonding. Similarly, the impact strength declined from 0.83 to 0.60 kJ/m^2^ (27.7% reduction) with 10 wt% Al, while Ti@Al incorporation elevated the impact resistance to 1.01 kJ/m^2^, representing a 20.5% enhancement, likely due to crack deflection and energy dissipation mechanisms facilitated by microcrack formation. Overall, this study demonstrates that while unmodified Al fillers enhance thermal conductivity, surface modification with titanate coupling agents provides a more balanced approach by improving mechanical properties and maintaining acceptable thermal performance. These insights highlight the importance of interfacial engineering in optimizing multifunctional composite materials for advanced thermal interface and electronic encapsulation applications.

## Figures and Tables

**Table 1:** Thermal conductivity of DGEBA/EG/Al and DGEBA/EG/Ti@Al composites.

Al Content (wt%)	Ti@Al Content (wt%)	Thermal Conductivity (W/(m⋅K))
0	0	7.35 ± 0.12
2.5	0	8.33 ± 0.13
5	0	8.76 ± 0.08
7.5	0	9.34 ± 0.10
10	0	9.60 ± 0.21
0	2.5	7.26 ± 0.26
0	5	6.68 ± 0.09
0	7.5	6.59 ± 0.08
0	10	6.80 ± 0.14

**Table 2:** Thermal stability factors of DGEBA/EG/Al and DGEBA/EG/Ti@Al composites obtained from TGA and DTA thermograms.

Al Content (wt%)	Ti@Al Content (wt%)	*T*_5%_ (°C) *^a^*	Amount of Char Formation at 600 °C (%) *^a^*
0	0	355.7	68.3
2.5	0	338.2	70.1
5	0	313.7	71.7
7.5	0	279.9	75.9
10	0	285.7	76.7
0	2.5	351.4	69.7
0	5	341.4	70.3
0	7.5	333.5	71.1
0	10	325.8	71.4

*^a^* Note: *T*5% and the amount of char formation at 600 °C were determined from TGA thermograms.

**Table 3:** Flexural strength of DGEBA/EG/Al and DGEBA/EG/Ti@Al composites.

Al Content (wt%)	Ti@Al Content (wt%)	Flexural Strength (MPa)
0	0	27.88 ± 0.61
2.5	0	26.74 ± 0.98
5	0	26.42 ± 1.36
7.5	0	20.51 ± 1.17
10	0	18.29 ± 1.27
0	2.5	33.71 ± 1.37
0	5	35.31 ± 1.46
0	7.5	34.91 ± 1.61
0	10	32.88 ± 1.51

**Table 4:** Impact strength of DGEBA/EG/Al and DGEBA/EG/Ti@Al composites.

Al Content (wt%)	Ti@Al Content (wt%)	Impact Strength (kJ/m^2^)
0	0	0.81 ± 0.02
2.5	0	0.80 ± 0.03
5	0	0.79 ± 0.01
7.5	0	0.62 ± 0.02
10	0	0.63 ± 0.04
0	2.5	0.82 ± 0.04
0	5	0.85 ± 0.04
0	7.5	0.94 ± 0.02
0	10	1.01 ± 0.04

## Data Availability

Data are contained within the article.
